# Natural Morin-Based Metal Organic Framework Nanoenzymes Modulate Articular Cavity Microenvironment to Alleviate Osteoarthritis

**DOI:** 10.34133/research.0068

**Published:** 2023-03-09

**Authors:** Jinhong Cai, Lian-feng Liu, Zainen Qin, Shuhan Liu, Yonglin Wang, Zhengrong Chen, Yi Yao, Li Zheng, Jinmin Zhao, Ming Gao

**Affiliations:** ^1^ Collaborative Innovation Centre of Regenerative Medicine and Medical Bioresource Development and Application Co-constructed by the Province and Ministry, The First Affiliated Hospital of Guangxi Medical University, Nanning, 530021, China.; ^2^ Guangxi Engineering Center in Biomedical Materials for Tissue and Organ Regeneration, Guangxi Key Laboratory of Regenerative Medicine, The First Affiliated Hospital of Guangxi Medical University, Nanning, 530021, China.; ^3^ Department of Ultrasound, Guangxi Medical University Cancer Hospital, Nanning, 530021, China.; ^4^ Department of Orthopedics, The First Affiliated Hospital of Guangxi Medical University, Nanning, 530021, China.; ^5^ Life Sciences Institute, Guangxi Medical University, Nanning, 530021, China.

## Abstract

Osteoarthritis (OA) is always characterized as excessive reactive oxygen species (ROS) inside articular cavity. Mimicking natural metalloenzymes with metal ions as the active centers, stable metal organic framework (MOF) formed by natural polyphenols and metal ions shows great potential in alleviating inflammatory diseases. Herein, a series of novel copper-morin-based MOF (CuMHs) with different molar ratios of Cu^2+^ and MH were employed to serve as ROS scavengers for OA therapy. As a result, CuMHs exhibited enhanced dispersion in aqueous solution, improved biocompatibility, and efficient ROS-scavenging ability compared to MH. On the basis of H_2_O_2_-stimulated chondrocytes, intracellular ROS levels were efficiently declined and cell death was prevented after treated by Cu_6_MH (Cu^2+^ and MH molar ratio of 6:1). Meanwhile, Cu_6_MH also exhibited efficient antioxidant and anti-inflammation function by down-regulating the expression of IL6, MMP13, and MMP3, and up-regulating cartilage specific gene expression as well. Importantly, Cu_6_MH could repair mitochondrial function by increasing mitochondrial membrane potential, reducing the accumulation of calcium ions, as well as promoting ATP content production. In OA joint model, intra-articular (IA) injected Cu_6_MH suppressed the progression of OA. It endowed that Cu_6_MH might be promising nanoenzymes for the prevention and treatment of various inflammatory diseases.

## Introduction

Osteoarthritis (OA) is a common joint degenerative disease caused by aging, obesity, strain, trauma and congenital joint abnormalities, etc. [1,2]. It is clinically manifested by progressive joint pain, swelling, stiffness, tenderness, and deformity, seriously affecting patients’ quality of life [3]. Moreover, reactive oxygen species (ROS) like hydroxyl radical (·OH), hydrogen peroxide (H_2_O_2_), and superoxide anion (·O_2_^−^) have been confirmed to be involved in OA pathogenesis by up-regulating the expression of inflammatory cytokines, leading to degeneration and destruction of extracellular matrix, and further contributing to OA progression. Meanwhile, excessive ROS can be markedly detected in joint cavity of OA patients, which even can reach 1 mM [4–6]. Therefore, ROS have been considered as a key marker of OA, and scavenging ROS can be applied as an effective strategy for OA therapy. Current studies have reported that ROS scavengers, such as melanin [7,8], vitamins [9], and nature polyphenols [10], have shown great potential in scavenging ROS and further alleviating OA severity. Nevertheless, most potent ROS scavengers have unrealistic biocompatibility and poor stability in joint cavity and are easy to be cleared by synovial fluid, limiting their further applications in clinical therapy of OA.

Morin hydrate (MH) is a natural active substance and contains many biological activities of flavonoids with low toxicity [11]. Numerous studies have shown that MH has good antioxidant and anti-inflammatory effects through ROS scavenging [12]. MH can prolong the survival of free radical-damaged cells and substantially possess better free radicals scavenging ability than that of vitamin, mannitol, and ascorbate [13]. It was widely applied in the treatment of mastitis [14], osteoporosis [15], osteoarthritis [16], and other inflammatory diseases. However, the therapeutic application of MH is severely limited by its insolubility, poor bioavailability, and rapid elimination from the organism. The nanosized preparation of MH is a possible method to solve these problems [17]. Recent evidences showed that stable metal organic framework (MOF) formed by natural herb-derived agents and metal ions could greatly improve the utilization rate and thus therapeutic effects, mimicking the coordination structures of natural metalloenzymes where the metals act as active centers [18]. With the introduction of active sites, electron transport interaction between drugs and metals endowed the novel agent with superior catalytic activity. It had been reported that epigallocatechin-3-gallate (EGCG), a natural polyphenol compound from green tea, could be chelated with Cu^2+^ [19], Sm^3+^ [20], or Mn^2+^ [21] to form a stable MOF, which could accelerate the repair of diseases like myocarditis, metastatic melanoma, and sepsis. Besides, tannic acid (TA), another kind of natural herb-derived agent, could be chelated with Ce^3+^ [22], Cu^2+^ [23,24], or Fe^3+^ [25,26] to form nanozymes with excellent catalytic activity, showing great potential in the therapy of inflammatory diseases. Compared to polyphenols like EGCG and TA itself, new formed MOF nanoenzymes could prolong the retention time in vivo. In addition, with slow release of EGCG under physiological environment, the drug utilization rate and therapeutic effect had been greatly improved [26]. Importantly, the effective combination of metal ions and EGCG or TA shielded the interaction between phenolic hydroxyl groups and led to more exposed phenolic hydroxyl groups, which were helpful to improving surface binding energy and ROS adsorption, further promoting antioxidant effect [23].

Among various metallic elements, Cu attracts the most attention because it plays a key role in various enzymatic reactions [27]. Accumulating evidence indicated that Cu nanoparticles were helpful to strengthen electron transfer reactions to shield the activity of ROS and achieve the function of ROS scavenging and ultimately improved skin, kidney, and liver injury treatment [28–30]. Importantly, Cu can also act as the connecting point by bridging ligands to form a stable MOF and plays its synergistic enhancement effect of antioxidation and anti-inflammation [24,31].

**Fig. 1.**
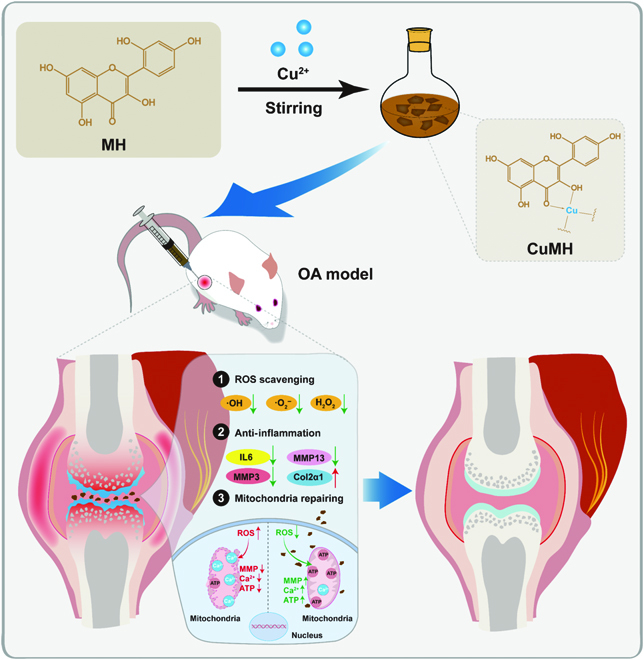
Schematic illustration of the preparation of natural polyphenols morin hydrate (MH)-based metal organic framework (CuMHs) by hydrothermal stirring and their in vivo therapy of OA rat model via modulating the microenvironment of articular cavity (ROS scavenging, anti-inflammation, and mitochondria repairing).

Inspired by MOF nanoenzymes with superior catalytic activities, we designed a series of CuMHs through the coordination of Cu^2+^ and MH as novel ROS scavengers to regulate the microenvironment of articular cavity for OA therapy (Fig. 1). Simultaneously, MH has multiple aromatic rings, standing out as a renewable source of rigid molecules. In addition, Cu, with various valences, acts as the connecting point for bridging MH. The novel designed CuMHs exhibited synergistic enhanced superoxide dismutase (SOD) and catalase (CAT)-like activities and ROS-scavenging capacities compared to MH alone. As revealed by H_2_O_2_-induced chondrocytes and OA joint models, CuMHs exhibited favorable biosafety and efficient ROS-scavenging ability to restore mitochondrial function, leading to the suppression of OA progression. Summarily, it might be employed as an effective method to fabricate natural polyphenol-based MOF with excellent ROS-scavenging capacity for the prevention and therapy of ROS-related diseases.

## Results and Discussion

### Basic characterization of CuMHs

**Fig. 2.**
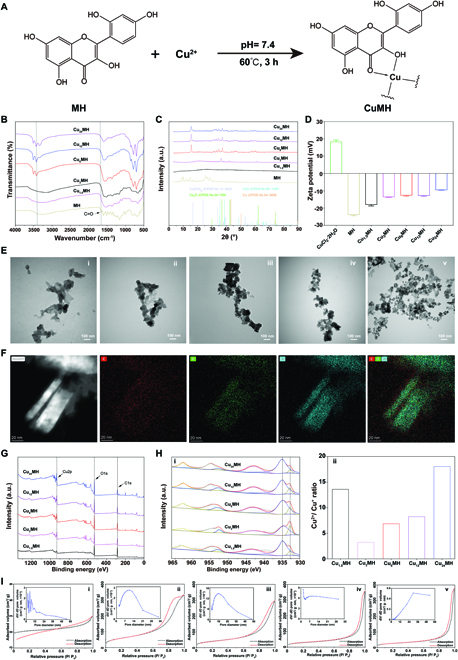
Preparation and characterization of CuMHs with different molar ratios of Cu^2+^ and MH. (A) Chemical synthesis of CuMHs. (B) FTIR results of MH and CuMHs. (C) XRD curves of MH and CuMHs. (D) Zeta potential of CuCl_2_·2H_2_O, MH, and CuMHs. (E) TEM images of Cu_1.5_MH (i), Cu_3_MH (ii), Cu_6_MH (iii), Cu_12_MH (iv), and Cu_24_MH (v). (Scale bar = 100 nm) (F) TEM-mapping images of Cu_6_MH. (Scale bar = 20 nm) (G) Full spectrum of CuMHs by XPS. (H) Detailed Cu spectrum of CuMHs (i) and the corresponding Cu^2+^/Cu^+^ ratio (ii) by XPS. (I) BET results of Cu_1.5_MH (i), Cu_3_MH (ii), Cu_6_MH (iii), Cu_12_MH (iv), and Cu_24_MH (v).

As a natural flavonoid compound with polyphenol groups, MH has attracted wide attention, while its biomedical use is often limited because of poor water solubility and low bioavailability. Therefore, transition metal element Cu was employed to coordinate with MH to obtain well-dispersed MH-based complexes: CuMHs. As shown in Fig. 2A, CuMHs were prepared by the coupling assembly of Cu^2+^ and MH with various molar ratios after thermal reflux reaction. MH has a strong chelating ability to form stable framework structures with multivalent metals. With a droplet of yellow MH solution, obvious crystals were observed, suggesting that the complexes were achieved between Cu^2+^ and MH. By adjusting the moles of Cu^2+^, different CuMHs were obtained and their corresponding symbols were Cu_1.5_MH, Cu_3_MH, Cu_6_MH, Cu_12_MH, and Cu_24_MH with a Cu^2+^ and MH molar ratio of 1.5:1, 3:1, 6:1, 12:1, and 24:1, respectively (Table S1). As shown in Fig. S1, the color of CuCl_2_·2H_2_O and MH was blue and brown, respectively. After forming complexes, the color became dark yellow for all CuMHs.

The molecular structure of CuMHs was firstly characterized by ultraviolet-visible (UV-vis) spectrum. From Fig. S2, 2 apparent peaks at 262 and 388 nm existed for MH, while no characteristic peaks were observed for CuCl_2_·2H_2_O. After forming CuMHs, 2 weak peaks still maintained at 245 and 290 nm. The shift of peaks was possibly attributed to the formation of CuMHs.

In addition, CuMHs were also characterized by Fourier transform infrared (FTIR). As illustrated in Fig. 2B, compared to MH alone, various characteristic peaks for CuMHs weakened or disappeared, possibly because of coupling assembly of Cu^2+^ and MH to shield various functional groups [21]. Specifically, the characteristic peak of a carbonyl group (C=O) of MH appeared at 1,657 cm^−1^, while it disappeared for CuMHs. In addition, the obvious broad peak (3,650 to 2,900 nm) was observed for MH, Cu_1.5_MH, and Cu_3_MH, while the splitting sharp peaks existed for Cu_6_MH, Cu_12_MH, and Cu_24_MH. The coordination between Cu^2+^ and –OH induced the disruption of –OH vibration [32], finally resulting in the existence of splitting peaks for CuMHs with the molar ratio of Cu^2+^ and MH above 3.

X-ray diffraction (XRD) is always used to evaluate the crystallization change of materials. Thus, the molecular structure of CuMHs was further characterized by XRD. As shown in Fig. 2C, it was observed that a few of obvious characteristic peaks appeared for MH below 30°, while no obvious diffraction peaks were observed for Cu_1.5_MH, indicating that it was an amorphous structure for Cu_1.5_MH. However, if the amount of Cu^2+^ during CuMHs preparation was increased, then the characteristic peaks gradually appeared for Cu_3_MH, Cu_6_MH, Cu_12_MH, and Cu_24_MH, demonstrating that the unique crystalline structure was formed for the above complexes. In particular, the obvious peak was observed at 34.6° for Cu_3_MH compared to other CuMHs, showing an intermediate transition state for Cu_3_MH. In the meantime, the unique characteristic peaks observed for Cu_6_MH, Cu_12_MH, and Cu_24_MH were also not similar with other Cu-based materials (e.g., Cu, Cu_2_O, CuO, or Cu(OH)_2_). Thus, when the molar ratio of Cu:MH was above 3, it was possible to fabricate CuMHs with appropriate crystalline structures.

To investigate the dispersion stability of CuMHs, their zeta potential was investigated. The zeta potential was −23.9 mV for MH due to the fact of a lot of –OH groups for MH itself. After forming the complex, the zeta potential of CuMHs obviously increased, attributed to the reason that the introduction of Cu^2+^ shielded –OH groups (Fig. 2D). In addition, the morphology of CuMHs was observed by transmission electron microscopy (TEM). As shown in Fig. 2E, the apparent irregular structure happened to Cu_1.5_MH and Cu_3_MH. However, with the increase of Cu^2+^ amount, it gradually changed to form a certain flake structure for Cu_6_MH, Cu_12_MH, and Cu_24_MH. Besides, to further confirm the three-dimensional structure of CuMHs, atomic force microscopy (AFM) was applied. As illustrated in Fig. S3, the height of Cu_6_MH was around 61.8 nm. Meanwhile, from the mapping images, it was obviously observed that only Cu, C, and O elements existed in Cu_6_MH (Fig. 2F).

Besides, X-ray photoelectron spectroscopy (XPS) was utilized to characterize the element composition and chemical state of CuMHs. From Fig. 2G, obvious Cu, C, and O elements were observed for CuMHs. The atomic ratio of Cu element markedly rose with the increase of Cu^2+^:MH molar ratio, which was 3.4% for Cu_1.5_MH and increased by 5.4-fold for Cu_24_MH. Conversely, C and O atomic ratios gradually declined with the increase of Cu^2+^:MH molar ratio (Table S3). Specifically, from fine spectra of Cu, it was clearly found that Cu^2+^ and Cu^+^ species existed in CuMHs. In addition, the fine spectra of Cu2p showed the obvious binding energies at 934.8 and 932.7 eV, respectively. (i of Fig. 2H) Specifically, for Cu_1.5_MH, the atomic ratio of Cu^2+^ and Cu^+^ was 93.1% and 6.9%, and Cu^2+^/Cu^+^ ratio was 13.6 due to its amorphous state. However, the Cu^2+^/Cu^+^ ratio was 3.2 for Cu_3_MH and increased by 2.1-, 2.5-, and 5.6-fold for Cu_6_MH, Cu_12_MH, and Cu_24_MH, respectively (ii of Fig. 2H). To our knowledge, the chelating Cu^2+^ could not be easily reduced by phenolic hydroxyl. Thus, it was little content of Cu^+^ for Cu_1.5_MH, leading to a high Cu^2+^/Cu^+^ ratio. Conversely, with the increase of Cu^2+^ dosage, more Cu^2+^ was exposed and reduced by phenolic hydroxyl, resulting in low Cu^2+^/Cu^+^ ratio for Cu_3_MH. If the dosage of Cu^2+^ continues to increase, then it also could increase Cu^2+^/Cu^+^ ratio. Thus, Cu^2+^/Cu^+^ ratio gradually rose with the increase of Cu^2+^ and MH molar ratio. Nevertheless, there were no obvious differences of fine spectra of C and O among different CuMHs (Fig. S4), indicating that different Cu^2+^:MH molar ratios could not affect the chemical state of C and O. After further calculation, the real Cu:MH molar ratio was listed in Table S1.

Furthermore, to confirm their MOF structures, Brunauer–Emmett–Teller (BET) assay was applied to explore the porosity of CuMHs. As indicated in Fig. 2I, it presented the adsorption–desorption isotherms of CuMHs. Because of extremely low specific surface area, the adsorption and desorption equilibrium of Cu_1.5_MH could not be established, indicating the formation of amorphous structures for Cu_1.5_MH. However, it displayed the typical adsorption–desorption isotherms with H3-type hysteresis for Cu_3_MH, Cu_6_MH, Cu_12_MH, and Cu_24_MH, respectively. Thus, the apparent porous structures were observed for CuMHs with the Cu^2+^:MH molar ratio above 3. After calculation, the pore volume and specific surface area was extremely low for Cu_1.5_MH. In addition, the order of pore volume was Cu_3_MH < Cu_6_MH < Cu_12_MH < Cu_24_MH, while it was in the order of Cu_3_MH > Cu_6_MH > Cu_12_MH > Cu_24_MH for specific surface area (Table S3). Combined the results of XRD and BET, it concluded that the stable MOF structures were established for Cu_6_MH, Cu_12_MH, and Cu_24_MH.

From the above, it confirmed the successful preparation of CuMHs with amorphous structure, intermediate state, and stable crystalline structure by adjusting the Cu^2+^:MH molar ratio. Significantly, when Cu:MH molar ratio was above 3, CuMHs could form a relative stable MOF with natural polyphenol MH as the ligand.

### In vitro degradation properties

For biomedical application, it was required that the materials possessed the degradation property without causing additional side effects after realizing their therapeutic effects. To evaluate its in vitro degradation property, Cu_1.5_MH and Cu_6_MH were immersed in 50 μM H_2_O_2_ (pH = 6.8) followed by investigating the concentration of Cu ions at predetermined time points (0, 3, 6, 12, 24, 48, and 72 h). In addition, 50 μM H_2_O_2_ (pH = 6.8) was applied to simulate the microenvironment of articular cavity of OA model with high ROS levels and weak acidic conditions [33]. From the results of Fig. S5, it was observed that the Cu ions release amount of Cu_1.5_MH was higher than that of Cu_6_MH. It also indicated that Cu_1.5_MH degraded relatively more quickly than the Cu_6_MH. The stability and degradability of materials are highly associated with their crystalline structures [34,35]. Because of its crystalline structure, it was relatively stable and not easy to be degraded for Cu_6_MH.

### ROS-scavenging ability

**Fig. 3.**
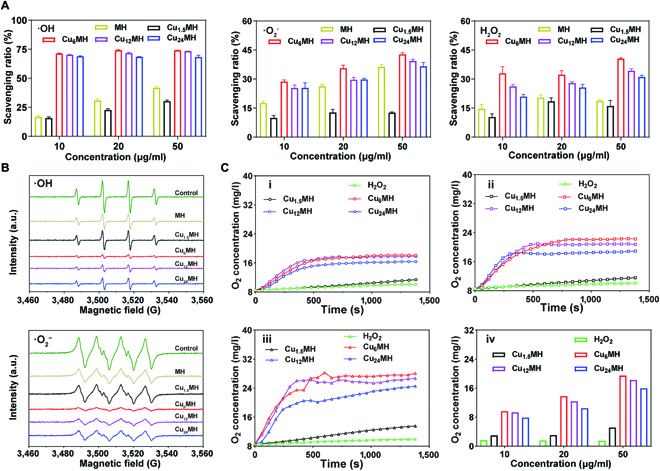
ROS-scavenging effect evaluation. (A) ·OH, ·O_2_^−^, and H_2_O_2_ scavenging ratio of MH and CuMHs with different concentrations (10, 20, and 50 μg/ml) by ROS testing kits. (B) ·OH and ·O_2_^−^ scavenging capacity of MH and CuMHs by ESR (20 μg/ml). (C) O_2_ release rate of 100 mM H_2_O_2_ incubated with CuMHs of different concentrations: 10 (i), 20 (ii), and 50 (iii) μg/ml and the corresponding quantification of O_2_ concentration (iv).

To explore ROS-scavenging capability of CuMHs, the ·OH, ·O_2_^−^, and H_2_O_2_ scavenging kits were applied. As shown in Fig. S6 and Fig. 3A, MH presented a certain of ·OH scavenging ability, still lower than that of CuMHs except for Cu_1.5_MH. Among all CuMHs, the order of ·OH scavenging ratio was Cu_6_MH > Cu_12_MH > Cu_24_MH > Cu_1.5_MH. In addition, increasing the amount of CuMHs, the corresponding ·O_2_^−^ scavenging ratios were also increased. The similar tendency was also observed for ·O_2_^−^ and H_2_O_2_ scavenging. It was observed that Cu_6_MH possessed the optimal scavenging ratios of ·O_2_^−^ and H_2_O_2_, followed by Cu_12_MH, Cu_24_MH, MH, and Cu_1.5_MH with the same concentrations. Specifically, if increasing the weight concentration of Cu_6_MH from 10 to 50 μg/ml, the corresponding scavenging effect increased by 1.5-fold for ·O_2_^−^ and 1.2-fold for H_2_O_2_, respectively (Fig. 3A). Besides, ROS-scavenging efficiencies were also evaluated by electron spin resonance (ESR). As illustrated in Fig. 3B, compared to the control group, all samples presented a certain of ROS-scavenging ability with obvious decreased signal observed. The order of ·OH and ·O_2_^−^ signal intensity was Cu_1.5_MH > MH > Cu_24_MH > Cu_12_MH > Cu_6_MH, indicating that the order of ·OH and ·O_2_^−^ scavenging capacity was Cu_1.5_MH < MH < Cu_24_MH < Cu_12_MH < Cu_6_MH. In the meantime, the O_2_ release rate was tested to assess H_2_O_2_ scavenging capacity by dissolved oxygen meter. It was obviously observed that O_2_ concentration maintained the baseline level while it rose slowly versus time for Cu_1.5_MH with different concentrations (10, 20, and 50 μg/ml). However, for Cu_6_MH, Cu_12_MH and Cu_24_MH, the O_2_ concentration markedly increased at the beginning and reached the equilibrium around 1,000 s (i, ii, and iii of Fig. 3C). After statistical calculation, it was possible to observe that the testing buffer could produce a certain concentration of O_2_ and it markedly increased for CuMHs with different concentrations. In addition, the order of O_2_ concentration was Cu_1.5_MH < Cu_24_MH < Cu_12_MH < Cu_6_MH. Among all groups, Cu_6_MH presented the optimum scavenging capacity of ·O_2_^−^, ·OH, and H_2_O_2_. The excellent ROS-scavenging capacity of Cu_6_MH was attributed to its high specific surface area and regular MOF structure [36,37]. Thus, Cu_6_MH was considered as artificial MOF nanoenzyme for further investigation.

### Biocompatibility in vitro

**Fig. 4.**
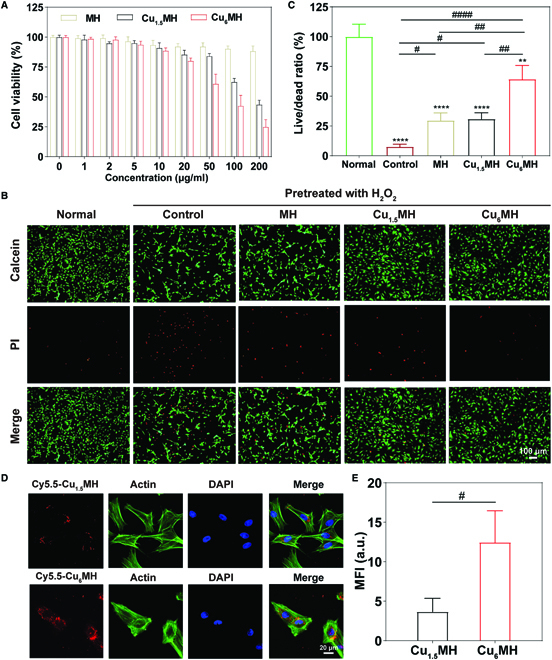
(A) Cell viability of MH, Cu_1.5_MH, and Cu_6_MH with the concentration ranging from 0 to 200 μg/ml by CCK-8 kit. (B) Live/dead staining of H_2_O_2_ (400 μM, 30 min)-induced chondrocytes after incubation by fluorescent microscopy, and the corresponding quantification of live/dead ratio (C). The samples were normal chondrocytes (Normal), H_2_O_2_-induced chondrocytes (Control), H_2_O_2_-induced chondrocytes after incubating with 20 μg/ml MH, Cu_1.5_MH, and Cu_6_MH, respectively. (D) Cellular uptake of Cy5.5-Cu_1.5_MH and Cy5.5-Cu_6_MH after 24 h by confocal microscopy (Cy5.5-Cu_1.5_MH or Cy5.5-Cu_6_MH: red, actin: green, and DAPI: blue) and the corresponding quantification (E). (“*” symbol compared with normal group, **P < 0.01, ***P < 0.001, and ****P < 0.0001, and “^#^” symbol compared between groups, ^#^*P* < 0.05, ^##^*P* < 0.01, ^###^*P* < 0.001, and ^####^*P* < 0.0001).

To determine chondrocyte cytotoxicity of CuMHs, cell counting kit-8 (CCK-8) assay was initially applied. As indicated in Fig. 4A, MH exhibited low cytotoxicity with the cell viability above 90% from 0 to 200 μg/ml. However, Cu_1.5_MH and Cu_6_MH exhibited a certain level of cytotoxicity with the increase of concentration due to the original cytotoxicity of Cu ions. The cell viability was above 80% for Cu_6_MH with the concentration of 20 μg/ml. Thus, 20 μg/ml was considered as the use concentration of all CuMHs for further experiments. Besides, to investigate their protection ability, the live/dead staining was applied by incubating H_2_O_2_-treated chondrocytes with 20 μg/ml CuMHs. From Fig. 4B, a lot of dead chondrocytes (red signal) were observed compared to normal chondrocytes. After incubation, the number of dead cells decreased for MH and Cu_15_MH, while only a few of dead cells were observed for Cu_6_MH. After statistical analysis, the live/dead ratio markedly decreased to 7.6% for chondrocytes after H_2_O_2_ stimulation compared to the normal group, and MH and Cu_1.5_MH improved the cell viability for a certain degree with increased live/dead ratio. Importantly, it was obviously observed that the live/dead ratio increased after Cu_6_MH treatment (Fig. 4C). Among all groups, Cu_6_MH possessed the optimum protection ability of chondrocytes away from the toxicity of H_2_O_2_, due to its excellent antioxidant ability.

### Cellular uptake

To give full play to their roles, it was also expected that nanoenzymes could be effectively uptake by the cells. Therefore, the nanoenzymes could scavenge not only the extracellular ROS but also intracellular ROS. As indicated in Fig. 4D, after 24 h of incubation, it was observed that fluorescent signal (red) existed inside the chondrocytes, indicating that Cy5.5-Cu_1.5_MH and Cy5.5-Cu_6_MH were taken up by the cells. Most importantly, it displayed higher fluorescence intensity for Cy5.5-Cu_6_MH than that of Cy5.5-Cu_1.5_MH (Fig. 4E). Therefore, the chondrocytes could take up more Cu_6_MH, leading to better therapeutic effect. Besides, the cells were also observed by TEM after 12 h. As illustrated by Fig. S6, Cu_6_MH was obviously observed inside the chondrocytes. In summary, it was possible for Cu_6_MH to be taken up by cells, finally to achieve its efficacy in intracellular levels.

### Antioxidant and anti-inflammatory capacity

**Fig. 5.**
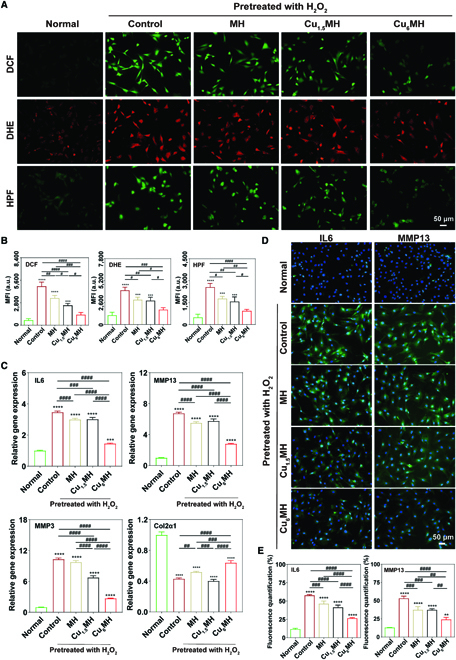
Antioxidant and anti-inflammatory capacity in cellular levels. (A) Intracellular ROS levels of H_2_O_2_ (400 μM, 30 min)-induced chondrocytes after incubation by fluorescent microscopy: total ROS levels (DCF), ·O_2_^−^ (DHE), and ·OH (HPF), and the corresponding quantification of mean fluorescent intensity (MFI) (B). (C) Genes (IL6, MMP13, MMP3, and Col2α1) expression of H_2_O_2_ (400 μM, 30 min)-induced chondrocytes after incubation by qRT-PCR. (D) Immunofluorescent images of IL6 and MMP13 expression of H_2_O_2_ (400 μM, 30 min)-induced chondrocytes after incubation by fluorescent microscopy and the corresponding quantification (E). The samples were normal chondrocytes (Normal), H_2_O_2_-induced chondrocytes (Control), and H_2_O_2-_induced chondrocytes after incubating with 20 μg/ml MH, Cu_1.5_MH and Cu_6_MH, respectively. (“*” symbol compared with normal group, **P < 0.01, ***P < 0.001, and ****P < 0.0001, and “^#^” symbol compared between groups, ^#^*P* < 0.05, ^##^*P* < 0.01, ^###^*P* < 0.001, and ^####^*P* < 0.0001).

To confirm the antioxidant capacity of CuMHs, intracellular ROS levels were evaluated by a ROS fluorescent probe. As illustrated in Fig. 5A, compared to normal chondrocytes, a lot of green fluorescence was observed for the control group by a 2′, 7′-dichlorofluorescin diacetate (DCF) probe, indicating high intracellular ROS levels after H_2_O_2_ stimulation. In addition, MH decreased the total ROS levels for a certain degree with slightly decreased green signal, while the green signal was significantly declined for Cu_6_MH, indicating high ROS-scavenging efficacy of Cu_6_MH. It also presented the same tendency for intracellular ·O_2_^−^ and ·OH levels by dihydroethidium (DHE) (red signal) and hydroxyphenyl fluorescein (HPF) (green signal) probes, respectively. Compared to normal chondrocytes, it presented high ·O_2_^−^ and ·OH levels for the control group with markedly increased fluorescent intensity, while Cu_6_MH presented close fluorescent intensity to the normal group, indicating the optimum ·O_2_^−^ and ·OH scavenging efficiencies. After statistical calculation, the mean fluorescent intensity (MFI) obviously increased for the control group after H_2_O_2_ stimulation compared to the normal group. Importantly, the MFI of Cu_6_MH was declined by 73.0%, 56.3%, and 63.3% compared to the control group for total ROS, ·O_2_^−^, and ·OH, respectively (Fig. 5B). From the above, H_2_O_2_ stimulation could increase the intracellular ROS levels, and Cu_6_MH presented the excellent scavenging ability of intracellular ROS.

Inflammation-induced degeneration was a typical feature of OA progression [38]. Thus, the inflammatory gene expression levels were quantified and analyzed by quantitative real-time polymerase chain reaction (qRT-PCR) to examine anti-inflammation ability of CuMHs. Particularly, interleukin 6 (IL6) played the key role in inducing matrix-degrading enzymes [39]. In addition, it was well known that matrix metalloproteinase 13 (MMP13) and MMP3 participated in cartilage degeneration and metastasis, and their up-regulation always resulted in the progression of cartilage degeneration [40]. Conversely, Col2α1 was an important constituent of extracellular matrix and possessed great necessity for cartilage regeneration [41]. From Fig. 5C, H_2_O_2_ stimulation markedly increased the levels of inflammation-related genes comparing with normal chondrocytes, where it was 3.5, 6.8, and 10.3 for IL6, MMP13, and MMP3, respectively, for the control group. Conversely, the expression of Col2α1, which was 0.4, was markedly reduced for the control group after H_2_O_2_ treatment. Most importantly, Cu_6_MH exhibited a significant decrease of the expression level of IL6, MMP13, and MMP3 compared to the control group, while there was a limited decrease for MH and Cu_1.5_MH. Meanwhile, Cu_6_MH could increase the expression of Col2α1 to some extent, while it did not show obvious changes for Cu_1.5_MH compared with the control group (Fig. 5C).

Besides, the inflammatory factors (IL6, MMP13, and MMP3) expression of cell supernatant was detected by enzyme-linked immunosorbent assay (ELISA). As shown in Fig. S8, all inflammatory factors markedly increased for chondrocytes after H_2_O_2_ stimulation. In addition, MH and Cu_1.5_MH slightly reduced their expression levels, in agreement with the results of qRT-PCR. However, it presented the similar condition that Cu_6_MH could most effectively reduce the expression of IL6, MMP13, and MMP3. The above results revealed that Cu_6_MH could reduce the expression levels of inflammation-related genes and promote the expression levels of chondrogenic gene, thus protecting cartilage to a certain extent.

Furthermore, the expression of inflammatory factors was also verified by immunofluorescent staining. As shown in Fig. 5D and E, after H_2_O_2_ treatment, the fluorescent intensity of the control group markedly increased more than that of normal chondrocytes, indicating an increased expression of IL6 and MMP13. However, MH, Cu_1.5_MH, and Cu_6_MH decreased the fluorescent intensity of H_2_O_2_-induced chondrocytes, especially for Cu_6_MH. Meanwhile, the IL6 and MMP13 expression levels increased by 389.9% and 308.4%, respectively, for the control group compared to normal chondrocytes. Importantly, Cu_6_MH greatly down-regulated IL6 and MMP13 expression by 54.0% and 54.5%, respectively. However, MH and Cu_1.5_MH only slightly lowered their expressions compared to the control group, showing similar results as those of qRT-PCR and ELISA.

Above all, these results showed that Cu_6_MH had superior antioxidant and anti-inflammation capacity, as well as could protect chondrocytes away from the attack of oxidative stress by maximally removing intracellular ROS, compared to MH and Cu_1.5_MH.

### Mitochondrial function repair

**Fig. 6.**
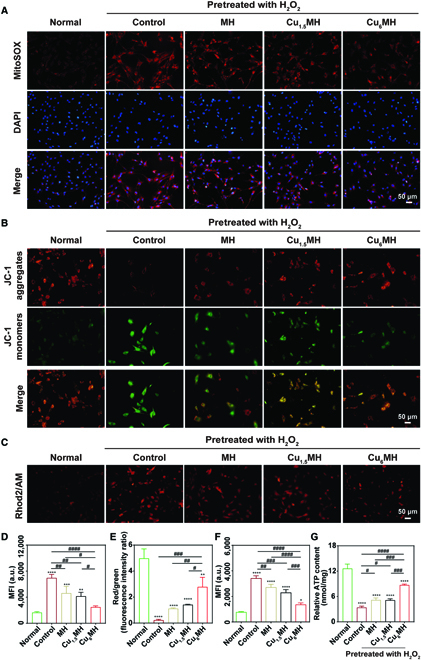
Mitochondrial function repair effects. (A) Endogenous ROS levels of H_2_O_2_ (400 μM, 30 min)-induced chondrocytes after incubation by fluorescent microscopy (MitoSOX: red and DAPI: blue), and the corresponding quantification (D). (B) Mitochondrial membrane potential of H_2_O_2_ (400 μM, 30 min)-induced chondrocytes after incubation by fluorescent microscopy (JC-1 aggregates: red and JC-1 monomers: green) and their corresponding quantification (E). (C) Mitochondrial Ca^2+^ level of H_2_O_2_ (400 μM, 30 min)- induced chondrocytes after incubation by fluorescent microscopy and the corresponding quantification (F). (G) ATP content of H_2_O_2_ (400 μM, 30 min)-induced chondrocytes after incubation. The samples were normal chondrocytes (Normal), H_2_O_2_-induced chondrocytes (Control), and H_2_O_2_-induced chondrocytes after incubating with 20 μg/ml MH, Cu_1.5_MH, and Cu_6_MH, respectively. (“*” symbol compared with normal group, *P < 0.05, **P < 0.01, ***P < 0.001, and ****P < 0.0001, and “^#^” symbol compared between groups, ^#^*P* < 0.05, ^##^*P* < 0.01, ^###^*P* < 0.001, and ^####^*P* < 0.0001).

ROS is mainly produced by mitochondria, and excessive ROS often lead to the damage of mitochondria [42,43]. Numerous studies confirmed that osteoarthritis was often caused by overproduced ROS [44,45]. Thus, scavenging endogenous ROS and repairing the function of mitochondria were helpful to achieving OA therapy with high efficacy. To confirm the above functions, the ROS levels inside mitochondria were assessed by a MitoSOX red mitochondrial superoxide indicator, which specifically targeted mitochondria to detect ·O_2_^−^ levels inside mitochondria. As shown in Fig. 6A, for normal chondrocytes, the ·O_2_^−^ levels were relatively low and almost no red signal was observed. After H_2_O_2_ stimulation, red signal markedly increased, indicating that high endogenous ROS levels appeared for the control group. In addition, it was observed that MH and Cu_1.5_MH could lower red signal to a certain level, equal to slightly decreasing the endogenous ROS levels. Nevertheless, Cu_6_MH markedly lowered the endogenous ROS levels with obvious decreased red signal observed. After statistical analysis, the MFI of the control group markedly increased compared to normal chondrocytes, while Cu_6_MH reduced the MFI by the most (Fig. 6D).

In the meantime, the membrane potential of mitochondria was also investigated by a JC-1 probe to reflect the function of mitochondria. The change of membrane potential was revealed by the transition between JC-1 aggregate (red) and JC-1 monomer (green) [46]. As indicated in Fig. 6B, a lot of red signal and little green signal were observed, meaning that a lot of JC-1 aggregates and few JC-1 monomers existed for normal chondrocytes; most of JC-1 aggregates disappeared and lots of JC-1 monomers appeared for normal chondrocytes after stimulation (control group). However, MH, Cu_1.5_MH, and Cu_6_MH increased the number of JC-1 aggregates and declined the amount of JC-1 monomers compared to the control group. After statistical calculation, the red/green ratio was 0.2 for the control group and increased by 5.0-, 6.3-, and 12.4-fold for MH, Cu_1.5_MH, and Cu_6_MH, respectively (Fig. 6E). Higher red/green ratio was equaled to higher mitochondrial membrane potential, indicating that more energy was produced by mitochondria, which promoted the energy conversion of cells. Thus, Cu_6_MH could most effectively reserve depolarization of mitochondrial membrane potential caused by oxidative stress compared to MH and Cu_1.5_MH, showing the excellent mitochondrial protection ability.

In addition, mitochondria plays a significant role in chondrocytes metabolism, participating in the regulation of cellular processes, such as redox homeostasis regulation and cellular Ca^2+^ balance [47]. Rhod2/Acetoxymethyl ester (AM), a high-affinity Ca^2+^ indicator excited by visible light, was applied to reflect the level of Ca^2+^ in cells. As indicated in Fig. 6C, compared to normal chondrocytes, lots of red signal was observed for the control group, indicating that Ca^2+^ level was markedly enhanced and Ca^2+^ balance was disordered for chondrocytes after H_2_O_2_ stimulation. Nevertheless, MH, Cu_1.5_MH, and Cu_6_MH could effectively decrease the Ca^2+^ level with reduced red signal, especially for Cu_6_MH. After statistical analysis, the order of MFI was normal group < Cu_6_MH < Cu_1.5_MH < MH < control group; corresponding to the order of Ca^2+^ level was control group > MH > Cu_1.5_MH > Cu_6_MH > normal group (Fig. 6F). It also confirmed that Ca^2+^ balance was redistributed after CuMHs or MH treatment, especially for Cu_6_MH.

Furthermore, adenosine triphosphate (ATP) is the main energy substance in cells and produced by mitochondria [48–50]. Thus, ATP content was applied to reflect the function of mitochondria. As shown in Fig. 6G, the relative ATP content was 12.6 nmol/mg for normal chondrocytes, which markedly decreased by 73.0% for the control group. Moreover, Cu_6_MH most effectively rescued the function of mitochondria with the relative ATP content of 8.7 nmol/mg, compared to MH and Cu_1.5_MH.

Above all, H_2_O_2_ stimulation markedly increased the endogenous ROS levels and Ca^2+^ level and reduced the mitochondrial membrane potential and relative ATP content. Among MH and CuMHs, Cu_6_MH presented the best effects of scavenging endogenous ROS, down-regulating Ca^2+^ level, improving mitochondrial membrane potential as well as promoting the ATP production, further protecting mitochondria away from oxidative damage, and activating the mitochondrial function of ROS-induced chondrocytes to achieve OA therapy.

### In vivo therapeutic effect

**Fig. 7.**
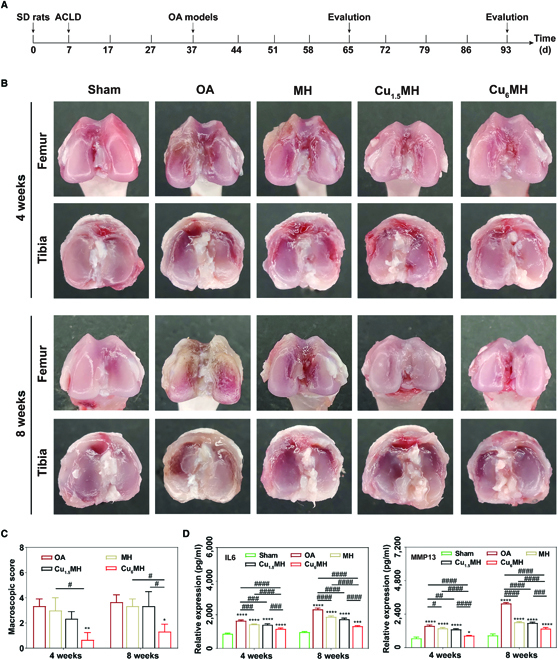
In vivo OA therapy effect. (A) Time schedule of in vivo experimental design. (B) Macroscopic observation of OA joints after IA injection at 4 and 8 weeks and their corresponding macroscopic scoring (C) (“*” symbol compared with OA group, *P < 0.05, **P < 0.01, and “#” symbol compared between groups, ^#^*P* < 0.05). (D) IL6 and MMP13 expression of joint fluid by ELISA. The corresponding groups were as follows: normal rats (Sham), OA rats by PBS treatment (OA), OA rats by MH treatment (MH), OA rats by Cu_1.5_MH treatment (Cu_1.5_MH), and OA rats by Cu_6_MH treatment (Cu_6_MH), respectively. (“*” symbol compared with sham group, *P < 0.05, **P < 0.01, ***P < 0.001, and ****P < 0.0001, and “^#^” symbol compared between groups, ^#^*P* < 0.05, ^##^*P* < 0.01, ^###^*P* < 0.001, and ^####^*P* < 0.0001) ACLD, anterior cruciate ligament-deficient.

To evaluate in vivo therapeutic effect, Sprague-Dawley rates were initially treated with anterior cruciate ligament-deficient surgery for 4 weeks followed by intra-articular (IA) injection of CuMHs (20 μg/ml, 100 μl) twice per week until another 4 weeks. The knee joints and synovial fluid of each rats were collected at 4 and 8 weeks to evaluate the therapeutic effect (Fig. 7A). The macroscopic observation of dissected knee joints was illustrated in Fig. 7B. From Fig. 7B, it was observed that the femur and tibia of articular surfaces were not damaged for the sham group and the corresponding cartilage surfaces were smooth. However, the obvious bone erosion and fissures appeared, and cartilage was deteriorated versus time for the OA group. In addition, the deterioration became weak for MH and Cu_1.5_MH at 4 weeks. Importantly, the apparent reduction of cartilage lesion and erosion existed for Cu_6_MH at 4 weeks. Meanwhile, the cartilage surface for MH and Cu_1.5_MH was uneven, with a certain of inflammation observed on the erosion area at 8 weeks. Specifically, Cu_6_MH markedly improved the inflammation degrees with no obvious erosion and defect areas existed. By Pelletier scoring, it was 3.3 at 4 weeks and 3.7 at 8 weeks, respectively, for the OA group, and it slightly decreased for MH and Cu_1.5_MH at both weeks. Most importantly, Cu_6_MH obviously reduced the scores by 80.0% at 4 weeks and 63.6% at 8 weeks, respectively, compared to the OA group, indicating the optimum OA therapeutic effect (Fig. 7C).

Next, the inflammatory factors expression of synovial fluid were also assessed to reflect OA therapeutic effects. As indicated in Fig. 7D, the IL6 and MMP13 expression was markedly ascended for the OA group compared with the sham group, confirming the successful establishment of OA models. In addition, MH and Cu_1.5_MH could reduce IL6 and MMP13 expression to some extent, with almost close variance analysis to OA group. However, their expressions were markedly declined for Cu_6_MH, decreased by 29.3% and 43.2% at 4 and 8 weeks for IL6 and by 44.7% and 57.1% at 4 and 8 weeks for MMP13, respectively. Meanwhile, the ROS levels of articular cartilage were also evaluated by a ROS testing kit. At 8 weeks, it increased by 12.0-fold for the OA group compared to the sham group, indicating high ROS levels in articular cartilage for OA rats. Notably, the ROS levels decreased by 63.5% for Cu_6_MH (Fig. S9). During OA progression, the inflammatory factors and ROS levels were always ascended versus time. However, Cu_6_MH could effectively reduce the inflammatory factors expression and ROS levels, corresponding to the optimum OA therapeutic effect.

**Fig. 8.**
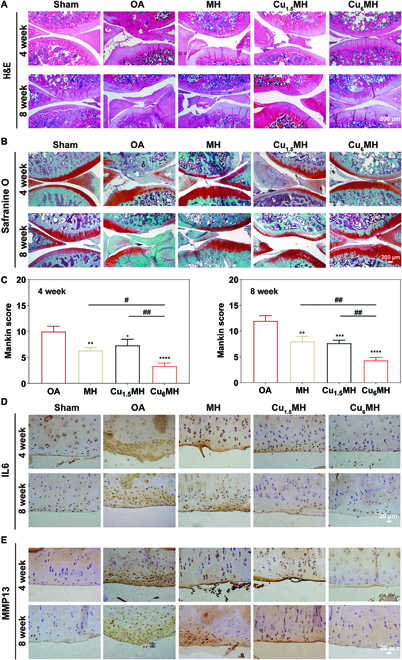
Histological evaluation of OA therapy. (A) H&E staining images of OA joints after therapy. (B) Safranine O-fast green staining images of OA joints after therapy and the corresponding Mankin scoring at 4 and 8 weeks (C). Immunohistochemical staining results of OA joints after treatment: IL6 (D) and MMP13 (E). The corresponding groups were as follows: normal rats (Sham), OA rats by PBS treatment (OA), OA rats by MH treatment (MH), OA rats by Cu_1.5_MH treatment (Cu_1.5_MH), and OA rats by Cu_6_MH treatment (Cu_6_MH). (“*” symbol compared with OA group, *P < 0.05, **P < 0.01, ***P < 0.001 and ****P < 0.0001, and “^#^” symbol compared between groups, ^#^*P* < 0.05 and ^##^*P* < 0.01).

Besides, hematoxylin and eosin (H&E) and Safranine O-fast green staining were applied to evaluate OA therapeutic effect. As illustrated in Fig. 8A, compared to the sham group, the obvious fissures, fibrillation, and matrix loss were observed for the OA group at 4 and 8 weeks. However, slight restoration of matrix happened to MH and Cu_1.5_MH. Especially for Cu_6_MH, it displayed obvious cartilage matrix restoration at both weeks. The similar tendency was also observed for Safranine O-fast green staining. For the OA group, thin and irregular cartilage layers were observed together with a lot of destroyed cartilage layers. However, the obvious restoration of cartilage layer and proteoglycan can appeared for Cu_6_MH, indicating the efficient OA therapy effect (Fig. 8B). After histological scoring, the Mankin score was 10.0 for the OA group, decreased by 37.0%, 27.0%, and 67.0%, respectively, for MH, Cu_1.5_MH, and Cu_6_MH at 4 weeks (Fig. 8C). Similarly, at 8 weeks, the Mankin score was 12.0, 8.0, 7.7, and 4.3, respectively, for the OA group, MH, Cu_1.5_MH, and Cu_6_MH (Fig. 8C). From the above, it revealed that Cu_6_MH displayed excellent cartilage protection on OA joints.

Furthermore, the immunohistochemical staining was applied to evaluate the expression of inflammatory factors further to reflect the anti-inflammatory effect. As shown in Fig. 8D, compared to the sham group, the brown staining markedly increased, indicating high expression of IL6 for the OA group. In the meantime, MH and Cu_1.5_MH could reduce the expression of IL6 to some extent with slightly decreased brown staining. However, apart from the sham group, the lowest positive staining was observed for Cu_6_MH, verifying the efficient in vivo anti-inflammatory effect. It also presented the same trend for the expression of MMP13 (Fig. 8E). The above results also indicated that Cu_6_MH optimally reduced the inflammatory factors expression and improved OA microenvironment, finally delaying the progression of OA.

Finally, the in vivo cytotoxicity was further evaluated by the histological analysis of major organs of Sprague-Dawley rats after treatment. From H&E staining of major organs, there were no obvious histopathological necrosis and inflammation lesions observed, close to the sham group, indicating no in vivo cytotoxicity during OA therapy (Fig. S7). Moreover, the content of Cu element in major organs was quantified detected by an inductively coupled plasma-mass spectrometer (ICP-MS). As illustrated in Fig. S8, the Cu contents in Cu_1.5_MH and Cu_6_MH groups were close to the sham group for all organs, meaning that Cu elements were removed from the rats and it would not cause additional cytotoxicity to major organs.

From all of the above, it had demonstrated that Cu_6_MH could effectively suppress the progression of OA with optimum effect. It was known to all that MH possessed a certain level of antioxidant and anti-inflammatory capacity, limited by its poor water solubility and low bioavailability. In addition, researches showed that Cu-based MOF could act as nanoenzymes with excellent ROS-scavenging ability [31]. However, it was expected that Cu-based MOF had favorable biodegradability so that it was possible to give full play to its ability of ROS scavenging for biomedical application. Thus, Cu_6_MH presented excellent capacity of scavenging both exogenous and endogenous ROS, and repairing the function of mitochondria, so as to regulate the microenvironment of articular cavity and further achieve most effectively protecting cartilage tissue and suppressing OA progression. In addition, it also could maintain relative stability and gradually degraded inside articular cavity, finally resulting in long-term effective OA therapy with no cytotoxicity.

## Conclusion

Avoid intrinsic cytotoxicity of nanoenzymes and bring into full play its ROS-scavenging activity are of high importance for the therapy of OA. Herein, the novel Cu_6_MH with Cu^2+^ and MH molar ratio of 6:1 was explored to act as nanoenzyme to alleviate OA. It had confirmed that Cu_6_MH could effectively scavenge ROS in all directions and activate the function of mitochondria, further regulating the microenvironment of articular cavity. The excellent antioxidant and anti-inflammatory capacity of Cu_6_MH indicated a very promising strategy for protecting articular cartilage and attenuating OA progression, leading to the remarkable OA therapeutic effects.

## Materials and Methods

### Materials

MH (>90%) and copper (II) chloride dihydrate (CuCl_2_·2H_2_O) were commercially obtained from Aladdin Biochemical Technology Co., Ltd (Shanghai, China). Sodium hydroxide (NaOH) was commercially purchased from Chron Chemical Co., Ltd (Chengdu, China). Hydrogen peroxide (H_2_O_2_, 30%) and ethanol were purchased from Sinopharm Chemical Reagent Co., Ltd (Shanghai, China). All reagents and chemicals were directly used without further treatment.

### Preparation of CuMHs

CuMHs were fabricated through the coupling assembly strategy as previously described [23,32]. In a typical synthesis, 0.04 g of MH and different amounts of CuCl_2_·2H_2_O were dissolved in 30 ml of mixed solution containing deionized (DI) water and ethanol with the volume ratio of 1:9. The mixture reacted at 60 °C for 3 h after adjusting pH = 7.4. The final products were obtained after centrifuge and DI water washing for 3 times, and finally vacuum drying overnight for further experiments. The detailed recipe for the preparation of CuMHs and their corresponding symbols are listed in Table S1.

### Basic characterization

The above prepared complexes were respectively characterized by using UV-vis spectroscopy (UV-2700, Shimadzu, Japan), FTIR spectrometer (IRAffinity-1S, Shimadzu, Japan) and XRD (MiniFlex 600, Japan). Their microstructure and element composition were also tested and analyzed by TEM (Hitachi, Japan) coupling with energy dispersive X-ray spectroscopy, and XPS (Thermo ESCALAB 250Xi, USA). Besides, their zeta potential in DI water was investigated by Nano-ZS (Malvern Panalytical, China). The three-dimensional morphology of samples was implemented by AFM (Bruker, USA). Finally, to evaluate the porosity of samples, the BET (Micromeritics ASAP, USA) was applied.

Meanwhile, the in vitro degradability of Cu_1.5_MH and Cu_6_MH was also evaluated. In brief, Cu_1.5_MH and Cu_6_MH (5 mg/ml) were immersed in 50 μM H_2_O_2_ (pH = 6.8) with a shaker (100 rpm, 37 °C) to simulate the OA microenvironment. The solutions at predetermined time points (0, 3, 6, 12, 24, 48, and 72 h) were collected and centrifuged at a high speed (10,000 rpm, 30 min). Cu concentration of the collected solutions were evaluated by using the dithizone chromogenic method (Macklin, China) according to the previous work with slight adjustment [51]. Briefly, 50 μl of sample solution was mixed with 600 μl of dimethyl sulfoxide (DMSO) solution containing 0.05% dithizone and 1% sodium acetate (Aladdin, China) in DMSO. After sufficiently mixing, 100 μl of solution was taken out and added to a 96-well plate, and the absorbance was detected by a microplate reader at 500 nm. Combing with Cu contents by ICP-MS, the Cu release rate of Cu_1.5_MH and Cu_6_MH was obtained.

### ROS-scavenging capacity investigation

The ROS-scavenging abilities of CuMHs were investigated by ·OH (Beijing baiaolaibo biotechnology, China), ·O_2_^−^ (Nanjing Jiancheng Bioengineering Institute, China) and H_2_O_2_ (Beyotime Biotechnology, China)-scavenging capacity assay kits, respectively after following the manufacturer’s instruction. Briefly, for ·OH scavenging ability, different concentrations of Cu_1.5_MH, Cu_6_MH, Cu_12_MH, Cu_24_MH, and MH (10, 20, and 50 μg/ml) were dispersed in the corresponding working solutions. The absorbance was observed at 550 nm by a microplate reader (Thermo Scientific, USA) after reacting for 1 min. Similarly, the absorbance was respectively obtained at 550 and 520 nm by using the microplate reader for investigating ·O_2_^−^ and H_2_O_2_ scavenging capacity.

Meanwhile, the ROS-scavenging effect of CuMHs was evaluated by ESR spectroscopy (ESR, Bruker A300, Germany) including ·OH and ·O_2_^−^. Briefly, 5-tert-butoxycarbonyl 5-methyl-1-pyrroline-N-oxide (BMPO) could react with ·OH and form stable adduct (BMPO/·OH) [52]. The signal of solution was recorded after mixing 5 mg/ml FeSO_4_, 100 mM BMPO, 10 M H_2_O_2_, and 20 μg/ml MH or CuMHs. Specifically, for ·O_2_^−^ testing, ·O_2_^−^ was initially generated by mixing xanthione (10 mM) and xanthione oxidase (1 U/ml) in phosphate buffer saline (PBS) buffer followed by the addition of 20 μg/ml MH or CuMHs. BMPO was applied to trap ·O_2_^−^ and form the spin adduct (BMPO/·OOH) [53].

Besides, O_2_ release concentration was determined by a dissolved oxygen meter (INSEA, China). In brief, different concentrations of CuMHs (10, 20, and 50 μg/ml) were added to 100 mM H_2_O_2_ and stirred for a certain of time. The data was recorded every 60 s by dissolved oxygen meter.

### Isolation and culture of chondrocytes

The femur and tibia from Sprague-Dawley rats (male, 3 to 5 d old) were applied to extract chondrocytes as previously described [40]. Briefly, the tissue was treated with Dulbecco’s modified eagle’s medium (Gibco, USA) containing 0.2% type II collagenase (Col2, Biofox, China) for a certain of time (6 h, 37 °C). And then, the undissociated substance was filtered by cell strainer (pore size of 0.45 μm). Chondrocytes were cultured with Dulbecco’s modified eagle’s medium containing 10% fetal bovine serum (FBS, Every Green, China), and 1% penicillin-streptomycin solution (Solarbio, China) in the incubator (37 °C, 5% CO_2_). At last, the medium was replaced every 2 days and the chondrocytes were passaged only 2 generations for further experiments.

### Cell viability evaluation

The cell viability of CuMHs was tested by CCK-8 (Biosharp, China) method. In detail, chondrocytes were cultured into 96-well plate (8,000 cells per well, 100 μl) and incubated for 24 h (37 °C, 5% CO_2_). The cultured medium was replaced with fresh medium containing different concentrations of MH or CuMHs (0, 1, 2, 5, 10, 20, 50, 100, and 200 μg/ml) respectively. After 24 h of incubation, chondrocytes were treated with fresh culture medium (100 μl, 10% CCK-8) after gently washing with PBS 3 times. After incubated for another 2 h, the absorbance was quantified measured (450 nm) by the microplate reader.

### Live/dead staining assay

Chondrocytes were seeded into 6-well plates (3 × 10^5^ cells per well) for further experiments. The inflammatory cell model was built by stimulating the chondrocytes with H_2_O_2_ solution (400 μM) for 30 min and then cocultured with 20 μg/ml MH, Cu_1.5_MH, or Cu_6_MH for another 24 h. Next, the cells were washed with PBS buffer 3 times followed by adding 500 μl of Calcein-AM/Propidium Iodide (AM/PI) staining solution (Beyotime Biotechnology, China) in the dark. Finally, the chondrocytes were washed again with PBS and photographed by using a fluorescent microscopy (OLYMPUS BX53F, Japan), and the number of live and dead cells was quantified by ImageJ.

### Intracellular ROS level testing

To verify intracellular ROS levels, chondrocytes were observed by fluorescent microscopy after treatment and staining. Details were as follows: The chondrocytes (2 × 10^5^ cells per well) were seeded in 6-well plates and incubated for 24 h. The chondrocytes were stimulated by H_2_O_2_ solution (400 μM) for 30 min before incubating with 20 μg/ml MH, Cu_1.5_MH, or Cu_6_MH overnight, respectively. Subsequently, the chondrocytes were respectively cocultured with 20 μM DCF (Solarbio, China) for total ROS testing, 10 μM HPF (maokangbio, China) for ·OH testing, or 5 μM DHE (Beyotime Biotechnology, China) for ·O_2_^−^ testing before PBS washing 3 times. Finally, the fluorescent intensity of chondrocytes was observed by fluorescent microscopy and the corresponding quantification was analyzed by ImageJ.

### Cellular uptake investigation

Chondrocytes were seeded in confocal dishes (1 × 10^5^ cells), and then incubated with Cy5.5-Cu_1.5_MH or Cy5.5-Cu_6_MH (20 μg/ml) in the dark for 24 h. After incubation, chondrocytes were washed with PBS 3 times and then fixed with paraformaldehyde (4% PFA, Biosharp, China) for 10 min. Later, the cytoskeleton was stained with the actin-tracker green-488 (Beyotime Biotechnology, China) following the protocols, and the nuclei was stained with 4, 6-diamidino-2-phenyindole dilactate (DAPI, Beyotime Biotechnology, China) for 10 min before PBS washing 3 times. Finally, the images were captured with a confocal scanning microscope (Leica, Germany), and the corresponding fluorescence intensity was quantified by ImageJ. Specifically, Cy5.5-Cu_1.5_MH and Cy5.5-Cu_6_MH were prepared by the following procedures. In detail, 50 mg of Cu_1.5_MH or Cu_6_MH was dispersed in DMSO followed by the addition of 50 mg/ml Cy5.5 N-hydroxysuccinimide ester (Cy5.5 NHS ester, Lumiprobe, USA) overnight. The final product was collected by vacuum drying after methanol washing. Besides, chondrocytes were incubated with Cu_6_MH (20 μg/ml) for 12 h, and chondrocytes were also embedded in paraffin and cut into slice for observation by TEM.

### Inflammatory factor evaluation

The inflammation factors expression levels of chondrocytes were initially investigated by qRT-PCR. Similarly, the chondrocytes were cultured into 6-well plates (2 × 10^5^ per well). After H_2_O_2_ (400 μM) stimulation for 30 min, the chondrocytes were treated with different samples (20 μg/ml) overnight. And then, total RNA of chondrocytes was extracted by an RNA extraction kit (Magen, China) following the protocols. The corresponding cDNA was synthesized according to reverse transcription kit (TaKaRa, China), and qRT-PCR was implemented with a LightCycler® system (Roche, Switzerland). Finally, the gene expression levels were analyzed by a 2^−ΔΔCT^ method and normalized with glyceraldehyde-3-phosphate dehydrogenase (GAPDH), and the corresponding detailed primer sequences are listed in Table S2. Besides, the supernatant of the corresponding chondrocytes was collected, and the expression levels of inflammatory factors (IL6, MMP13, and MMP3) were also detected using ELISA kits (Meinian, China) according to the manufacturer’s instructions.

To further verify IL6 and MMP13 expression levels, the treated chondrocytes (2 × 10^5^ per well) were fixed with PFA (4%, 15 min), stimulated with 3% H_2_O_2_, and blocked with bovine serum albumin working solution (ZS-Bio, China) for 15 min. Later, chondrocytes were stained against the antibody of anti-IL6 or anti-MMP13 (1:200 dilution, Bioss, China) overnight after washing with PBS buffer 3 times. Next, the cells were reacted against the specific fluorescein isothiocyanate-labeled secondary antibody (1:100 dilution, Boster, China) in the dark (37 °C, 1 h) before PBS buffer washing. Meanwhile, the nuclei of cells was costained using DAPI in the dark (15 min). Finally, the images were captured by fluorescent microscopy, and their corresponding quantification was analyzed by ImageJ.

### Endogenous ROS levels

The endogenous ROS levels from mitochondria were evaluated by MitoSOX red mitochondrial superoxide indicator (Yeason, China) by following the procedure of manufacture. In brief, chondrocytes were seeded in 6-well plates (2 × 10^5^ per well) and stimulated with H_2_O_2_ (400 μM) for 30 min. Chondrocytes were cocultured with different types of samples (20 μg/ml, 24 h) before PBS washing. After incubation with MitoSOX (5 μM, 30 min) at 37 °C, chondrocytes were washed with PBS another 3 times. And then, the cells were fixed with 4% PFA, and the nuclei was stained with DAPI solution (15 min). Ultimately, the images were photographed by fluorescent microscopy and their corresponding intensity was quantified analyzed by ImageJ.

### Mitochondrial membrane potential

The JC-1 probe (Beyotime Biotechnology, China) was applied to evaluate the mitochondrial membrane potential of chondrocytes. In brief, chondrocytes (2 × 10^5^ per well) were treated with 400 μM H_2_O_2_ for 30 min followed by incubating with samples (20 μg/ml) for another 24 h. After incubating with JC-1 probe (20 min, 37 °C), the mitochondrial membrane potential was observed using by fluorescent microscopy, and the red/green fluorescence ratio was analyzed by ImageJ.

### Mitochondrial calcium levels

The mitochondrial calcium levels were investigated by Rhod2/AM cell permeable calcium ion (Ca^2+^) fluorescent probe (Yeasen, China). In brief, H_2_O_2_ (400 μM, 30 min) treated chondrocytes (3 × 10^4^ per well) were incubated with different kinds of samples (20 μg/ml) for 24 h and stained with Rhod2/AM probe (4 μM, 0.02% Pluronic F127, Sigma, USA) at 37 °C for another 30 min. After PBS washing for 3 times, the images were obtained by fluorescent microscopy and the corresponding fluorescent intensity was quantified analyzed by ImageJ.

### ATP production

The ATP content of chondrocytes was tested by an ATP content detection kit (Beyotime biotechnology, China). In detail, chondrocytes (3 × 10^5^ per well) were treated with 400 μM H_2_O_2_ (30 min) and then cocultured with samples (20 μg/ml) for another 24 h. The supernatant was saved and tested by the microplate reader (BioTek, USA) following the protocol. In order to eliminate errors of protein concentration, the final results were denoted as nM/mg protein based on a BCA protein quantification kit (Beyotime biotechnology, China).

### Establishment of OA models

Sprague-Dawley rats (male, 150~200g) were obtained from Guangxi Medical University Experimental Animal Center, and all experiments were implemented in accordance with institutional guidelines and approved by Animal Ethics Committee of Guangxi Medical University. The OA model was established by anterior cruciate ligament-deficient surgery of Sprague-Dawley rats and fed for 1 month before further experiments. The rats were separately divided into 5 groups: (1) Sham: rats were only treated with incising the joint capsule, (2) OA: OA rats with PBS injection (100 μl), (3) MH: OA rats with MH injection (20 μg/ml, 100 μl), (4) Cu_1.5_MH: OA rats with Cu_1.5_MH injection (20 μg/ml, 100 μl), and (5) Cu_6_MH: OA rats with Cu_6_MH injection (20 μg/ml, 100 μl). The rats were treated with IA injection twice per week until 4 or 8 weeks before overdose of pentobarbital sodium, and the corresponding knee joints and other major organs were collected for further analysis. Specifically, the knee joints were imaged and evaluated by Pelletier’s macroscopic scoring.

### Inflammatory factors expression of synovial fluid

IL6 and MMP13 expression of joint fluid was measured by ELISA kits by following the protocols. The corresponding absorbance was obtained by the microplate reader at 450 nm.

### ROS levels of articular cartilage

The ROS levels of articular cartilage were measured by a tissue ROS detection kit (Bestbio, China) according to the manufacturer’s instruction. In brief, 50 mg of cartilage tissue was made into homogenate with 1 ml of PBS buffer, and the supernatant of homogenate was centrifuged (1,000 rpm, 4 °C) for 3 min and collected for further analysis. Then, 1 μl of ROS probe (BBoxiProbe) was added to 200 μl of collected solutions, and the mixture was incubated at 37 °C for 30 min. Finally, the absorbance of mixture was detected by the microplate reader (BioTek, USA) at an excitation wavelength of 488 nm and an emission wavelength of 610 nm.

### Histological staining of joint section

After euthanasia at 4 and 8 weeks, the knee joins were fixed with 4% PFA and decalcified in ethylene diamine tetra acetic acid (EDTA, pH = 7.2, Macklin, China) by Ultrasonic Decalcifying Unit (USE 33, Germany) for 1 month. The joint samples were embedded and sectioned in paraffin (5-μm thickness) for further staining. The joint sections were respectively stained with H&E (Solarbio, China) and Safranine O-fast green (Safranine O, Solarbio, China) for histological analysis, and OA progression was evaluated according to Mankin scoring as previously described by 2 blinded observers [41]. Specifically, for immunohistochemical staining, the sections were initially incubated with rabbit polyclonal anti-IL6 or anti-MMP13 (Boster, China) antibodies overnight at 4 °C followed by binding with biotinylated secondary antibodies (ZS-Bio, China). The slides were photographed by optical microscopy (OLYMPUS BX53F, Japan).

### In vivo cytotoxicity evaluation

Major organs including heart, liver, spleen, lung, and kidney from Sprague-Dawley rats were initially fixed in 4% PFA for 2 days and processed as paraffin sections followed by H&E staining. Besides, the Cu^2+^ contents of each organs were quantified analyzed by ICP-MS (Varian, 720-ES, USA).

### Statistical analysis

All data were expressed as means ± SD and analyzed by one-way analysis variance (ANOVA) or Student *t* test for comparisons by GraphPad Prism 8.0. The differences with *P* < 0.05 indicate significance.

## Data Availability

All data needed to evaluate the conclusions in the paper are present in the paper and/or the Supplementary Materials. Additional data related to this paper may be requested from the authors.

## Supplementary Materials

Supplementary Materials Fig. S1. Optical image of CuCl_2_·2H_2_O, MH, and CuMHs. Fig. S2. UV-vis spectrum of CuCl_2_·2H_2_O, MH, and CuMHs. Fig. S3. AFM image of Cu_6_MH. Fig. S4. Detailed C and O spectrum of CuMHs by XPS. Fig. S5. Degradability of Cu_1.5_MH and Cu_6_MH. Fig. S6. Images of ·OH, ·O_2_^−^, and H_2_O_2_ scavenging ability testing after incubating with different concentrations of MH and CuMHs (10, 20, and 50 μg/ml). Fig. S7. Cellular uptake of Cu_6_MH at 12 h by TEM. Fig. S8. Inflammatory factors (IL6 (i), MMP13 (ii), and MMP3 (iii)) expression of the supernatant of H_2_O_2_-induced chondrocytes after treatment by ELISA. Fig. S9. ROS levels inside articular cavity of Sprague-Dawley rats after treatment at 8 weeks. Fig. S10. H&E staining images of major organs including heart, liver, spleen, lung, and kidney of Sprague-Dawley rats after treatment at 8 weeks. Fig. S11. Cu element contents of major organs including heart, liver, spleen, lung, and kidney of Sprague-Dawley rats after treatment at 8 weeks. Table S1. Detailed recipe for the preparation of CuMHs. Table S2. Detailed primer sequences for qRT-PCR. Table S3. C, O, and Cu contents of CuMHs by XPS and the corresponding surface adsorption properties by BET.
