# Supplementary material for: Natural Morin-Based Metal Organic Framework Nanoenzymes Modulate Articular Cavity Microenvironment to Alleviate Osteoarthritis
Source: Research (Wash D C). 2023 Mar 9;6:0068. doi: 10.34133/research.0068 (PMC10013961; doi:10.34133/research.0068)
Supplement: Supplementary Materials — Fig. S1. Optical image of CuCl2·2H2O, MH, and CuMHs. Fig. S2. UV-vis spectrum of CuCl2·2H2O, MH, and CuMHs. Fig. S3. AFM image of Cu6MH. Fig. S4. Detailed C and O spectrum of CuMHs by XPS. Fig. S5. Degradability of Cu1.5MH and Cu6MH. Fig. S6. Images of ·OH, ·O2−, and H2O2 scavenging ability testing after incubating with different concentrations of MH and CuMHs (10, 20, and 50 μg/ml). Fig. S7. Cellular uptake of Cu6MH at 12 h by TEM. Fig. S8. Inflammatory factors (IL6 (i), MMP13 (ii), and MMP3 (iii)) expression of the supernatant of H2O2-induced chondrocytes after treatment by ELISA. Fig. S9. ROS levels inside articular cavity of Sprague-Dawley rats after treatment at 8 weeks. Fig. S10. H&E staining images of major organs including heart, liver, spleen, lung, and kidney of Sprague-Dawley rats after treatment at 8 weeks. Fig. S11. Cu element contents of major organs including heart, liver, spleen, lung, and kidney of Sprague-Dawley rats after treatment at 8 weeks. Table S1. Detailed recipe for the preparation of CuMHs. Table S2. Detailed primer sequences for qRT-PCR. Table S3. C, O, and Cu contents of CuMHs by XPS and the corresponding surface adsorption properties by BET. [file research.0068.f1.docx]

**Supplemental Materials**

**Natural morin based metal organic framework nanoenzymes modulate articular cavity microenvironment to alleviate osteoarthritis**

Jinhong Cai^1,2†^, Lian-feng Liu^1,3†^, Zainen Qin^1,2†^, Shuhan Liu^1,2^, Yonglin Wang^1,4^, Zhengrong Chen^2,5^, Yi Yao^2,5^, Li Zheng^1,2*^, Jinmin Zhao^1,2,4*^, and Ming Gao^1,2*^


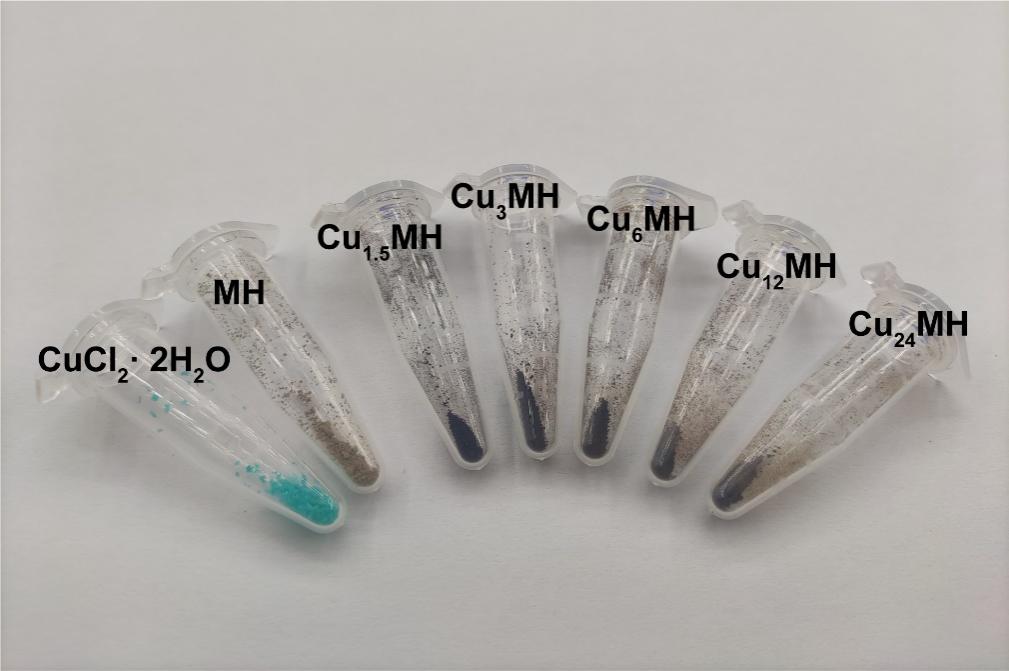


Fig. S1. Optical image of CuCl_2_·2H_2_O, MH and CuMHs.


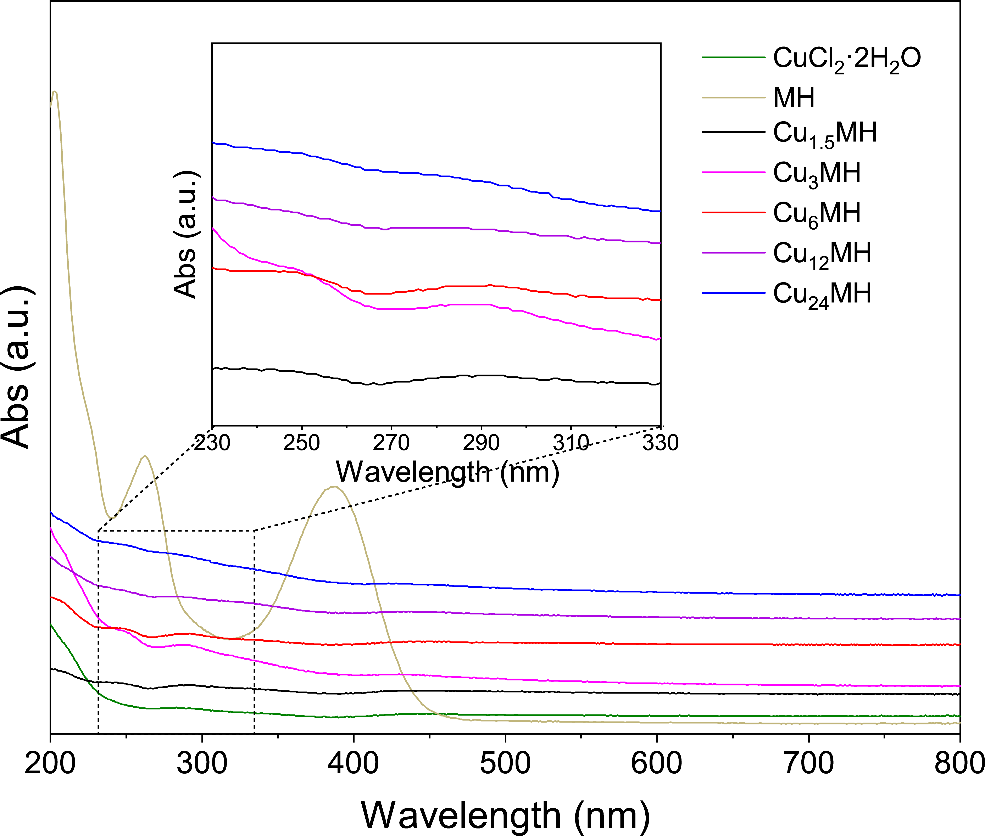


Fig. S2. UV- vis spectrum of CuCl_2_·2H_2_O, MH and CuMHs.


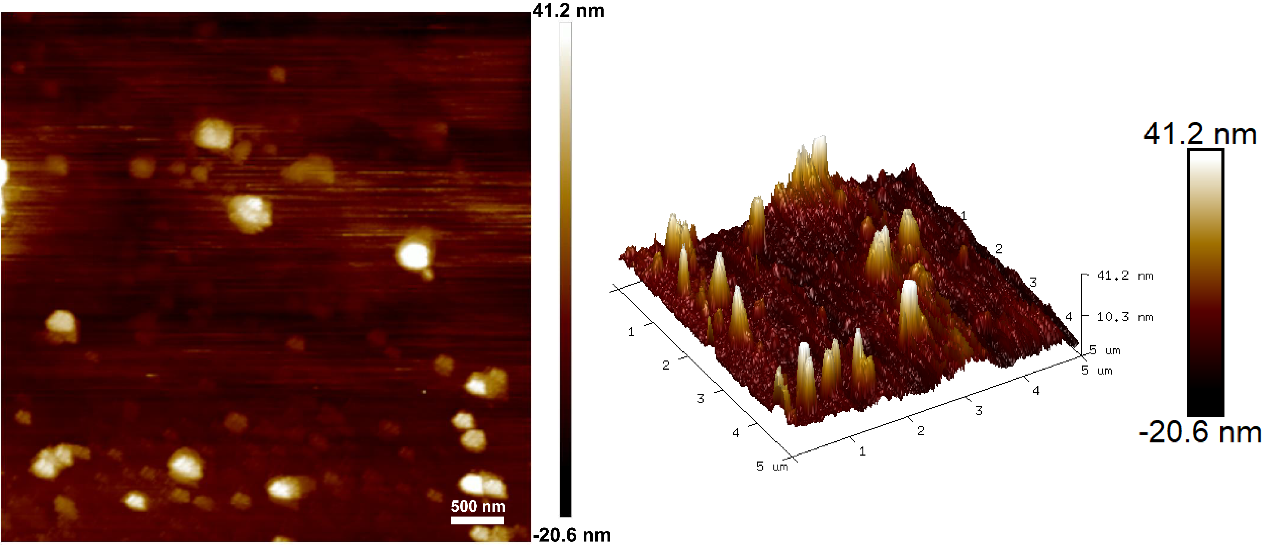


Fig. S3. AFM image of Cu_6_MH.


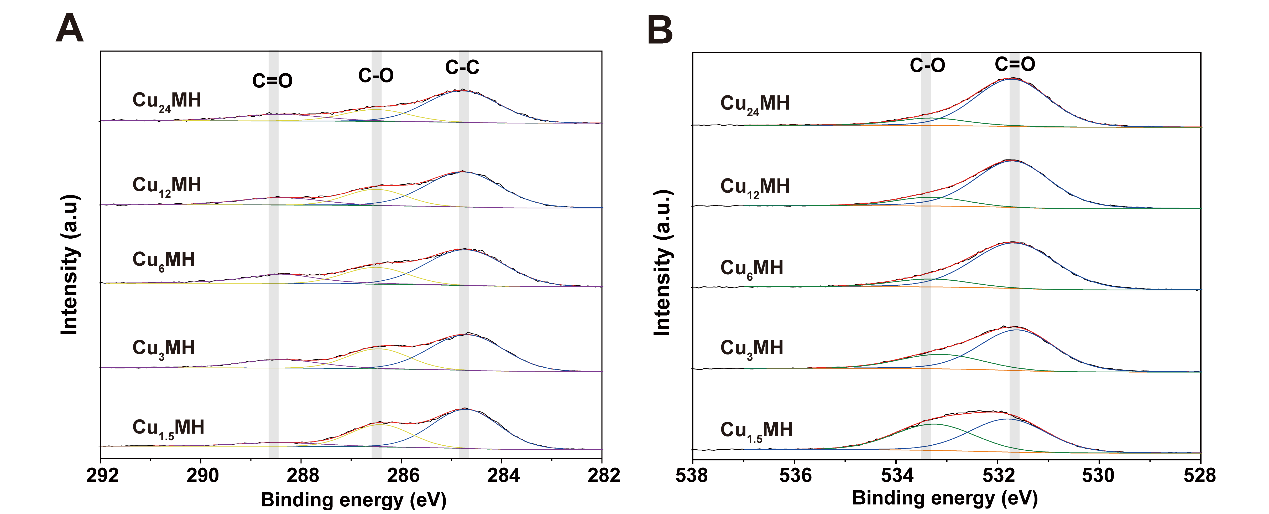


Fig. S4. Detailed C and O spectrum of CuMHs by XPS.


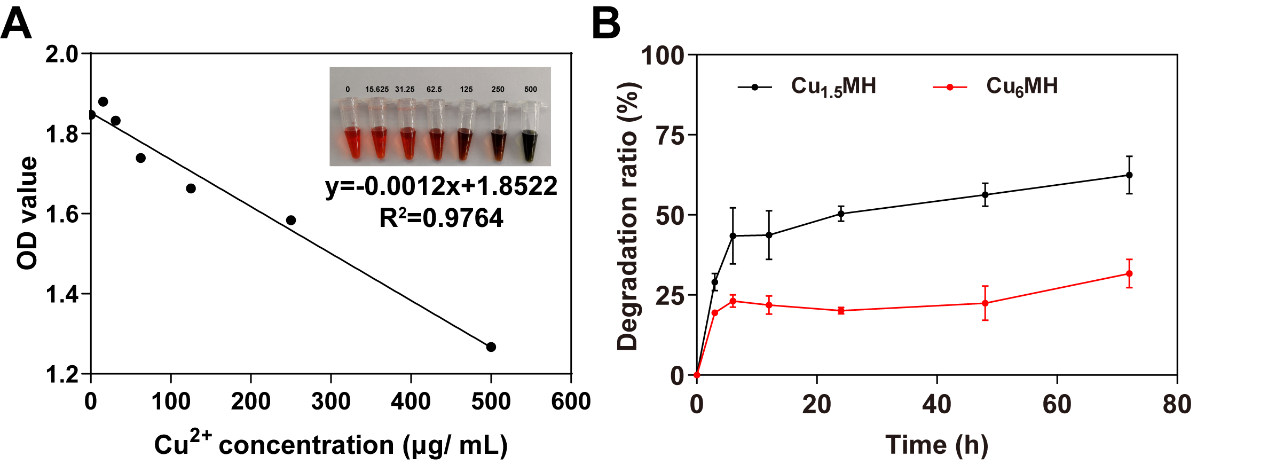


Fig. S5. Degradability of Cu_1.5_MH and Cu_6_MH. A) The standard curve of Cu ions by UV-vis spectrum. B) Cu ions release rate of Cu_1.5_MH and Cu_6_MH in 50 μM H_2_O_2_ (pH= 6.8).


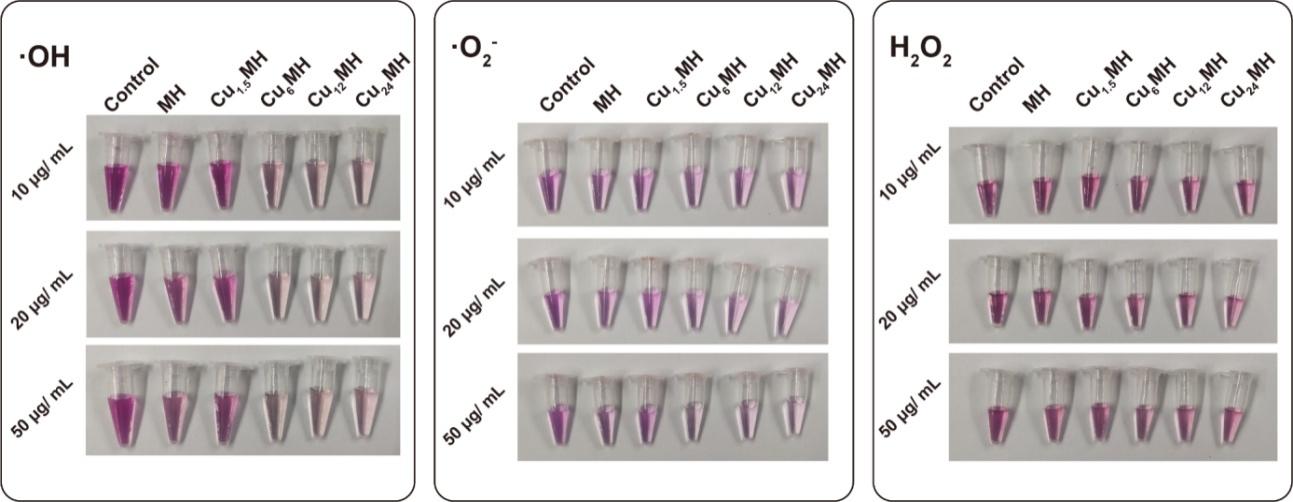


Fig. S6. Images of ·OH, ·O_2_^-^ and H_2_O_2_ scavenging ability testing after incubating with different concerntrations of MH and CuMHs (10, 20 and 50 μg/ mL).


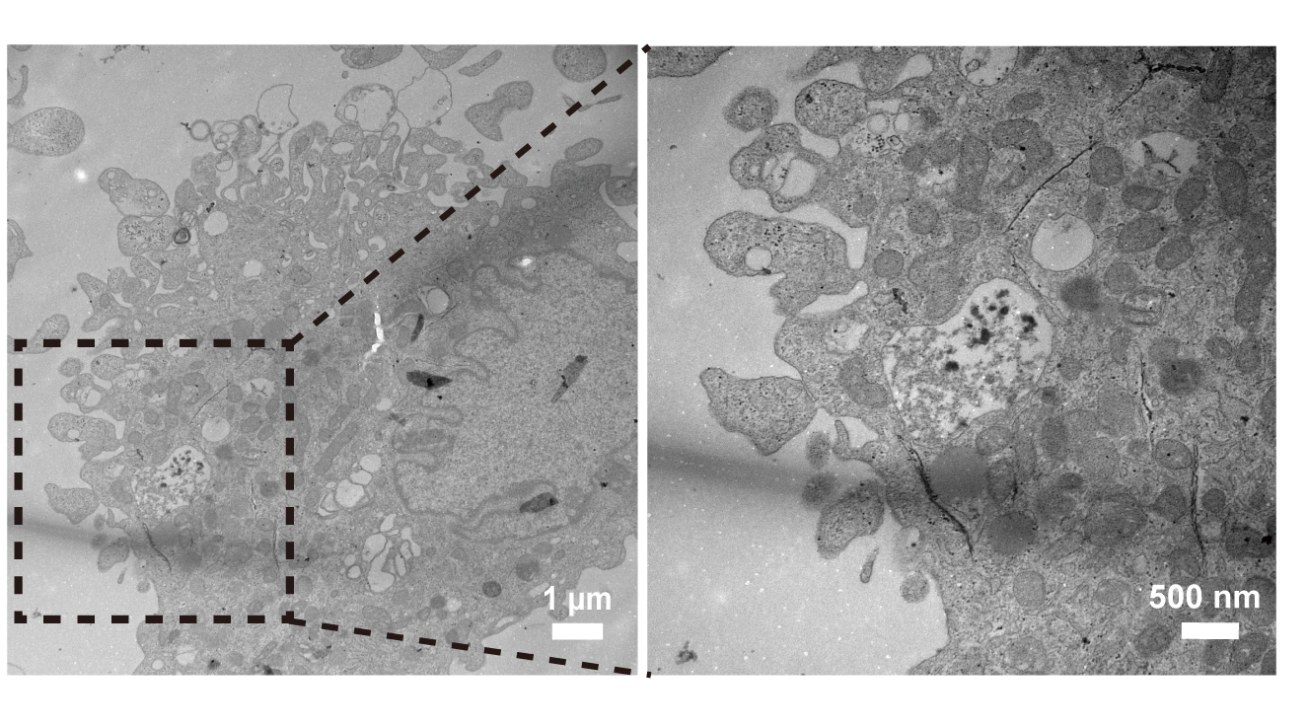


Fig. S7. Cellular uptake of Cu_6_MH at 12 h by TEM.


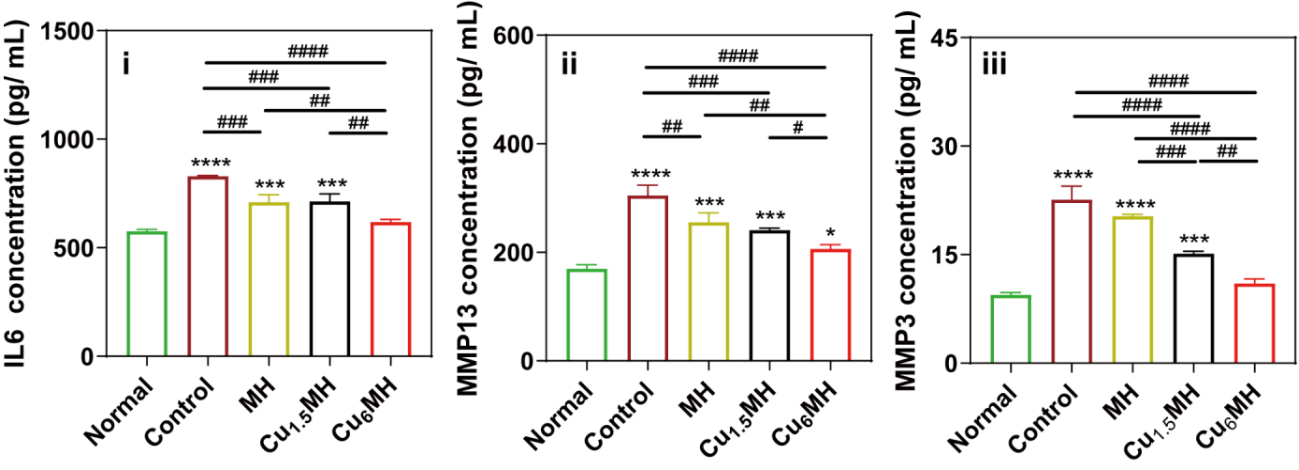


Fig. S8. Inflammatory factors (IL6 (i), MMP13 (ii) and MMP3 (iii)) expression of the supernatant of H_2_O_2_ induced chondrocytes after treatment by ELISA. Samples were normal chondrocytes (Normal), H_2_O_2_ induced chondrocytes (Control), and H_2_O_2_ induced chondrocytes after incubating with 20 μg/ mL MH, Cu_1.5_MH and Cu_6_MH respectively. (‘*’ symbol compared with normal group, *p< 0.05, **p< 0.01, ***p< 0.001 and ****p< 0.0001, and ‘^#^’ symbol compared between groups, ^#^p< 0.05, ^##^p< 0.01, ^###^p< 0.001 and ^####^p< 0.0001)


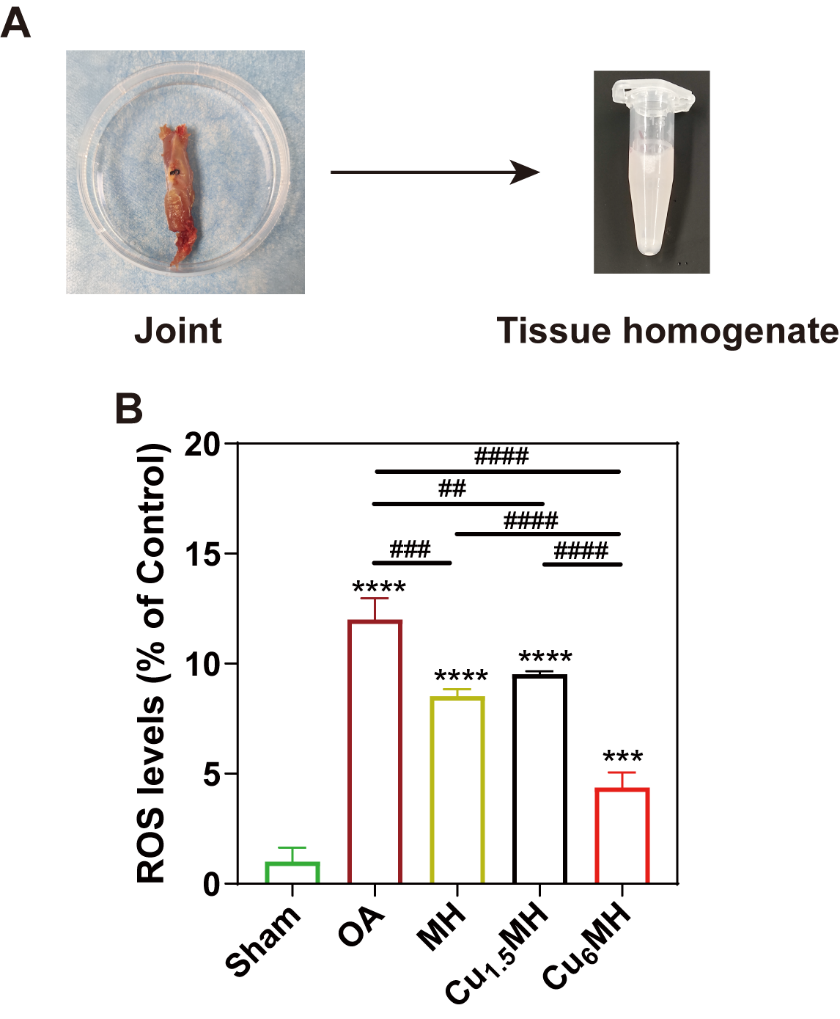


Fig. S9. ROS levels inside articular cavity of SD rats after treatment at 8 week. A) Comparison of cartilage tissue homogenate before and after extraction. B) Relative ROS levels inside articular cavity by ROS testing kit. The corresponding groups were: normal rats (Sham), OA rats (OA), OA rats by MH treatment (MH), OA rats by Cu_1.5_MH treatment (Cu_1.5_MH), and OA rats by Cu_6_MH treatment (Cu_6_MH) respectively. (‘*’ symbol compared with sham group, ***p< 0.001 and ****p< 0.0001, and ‘^#^’ symbol compared between groups, ^##^p< 0.01, ^###^p< 0.001 and ^####^p< 0.0001)


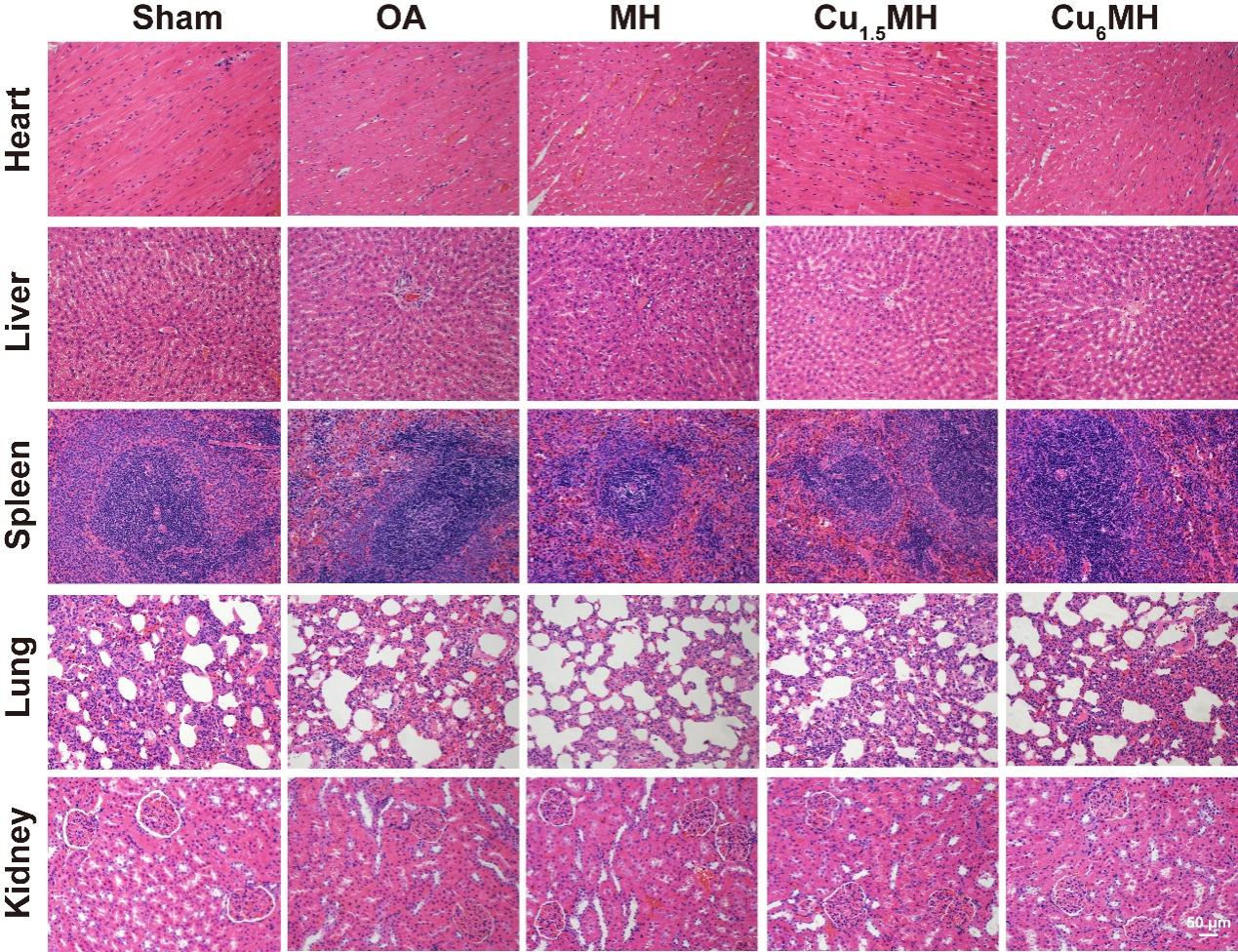


Fig. S10. H&E staining images of major organs including heart, liver, spleen, lung and kidney of SD rats after treatment at 8 week. The corresponding groups were: normal rats (Sham), OA rats (OA), OA rats by MH treatment (MH), OA rats by Cu_1.5_MH treatment (Cu_1.5_MH), and OA rats by Cu_6_MH treatment (Cu_6_MH) respectively.


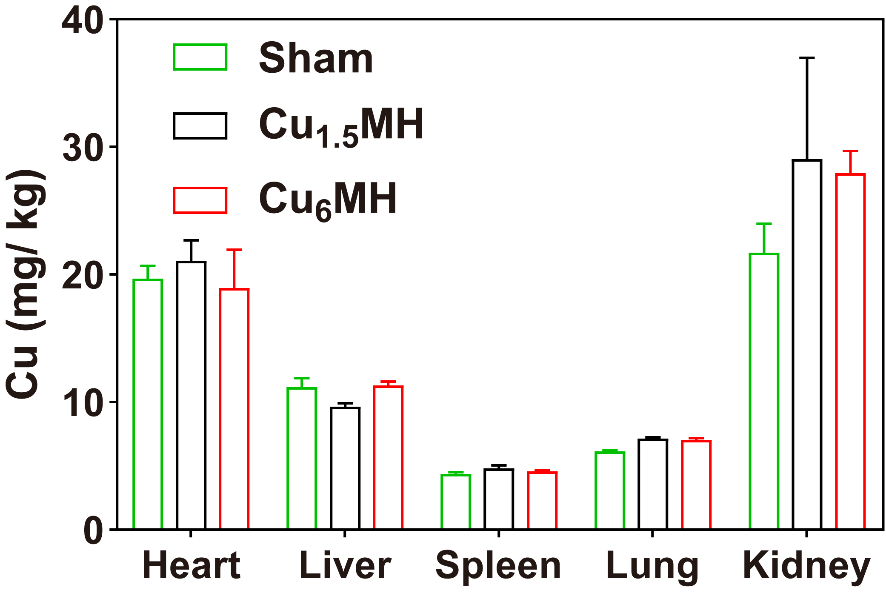


Fig. S11. Cu element contents of major organs including heart, liver, spleen, lung and kidney of SD rats after treatment at 8 week. The corresponding groups were: normal rats (Sham), OA rats by Cu_1.5_MH treatment (Cu_1.5_MH), and OA rats by Cu_6_MH treatment (Cu_6_MH) respectively.

Table S1. Detailed recipe for the preparation of CuMHs.

| Symbol | CuCl_2_·2H_2_O (g) | Morin hydrate (MH, g) | Molar ratio of Cu^2+^ and MH | |
| --- | --- | --- | --- | --- |
|  |  |  | Feeding ratio | Real ratio |
| Cu_1.5_MH | 0.034 | 0.04 | 1.5: 1 | 1.11: 1 |
| Cu_3_MH | 0.068 | 0.04 | 3.0: 1 | 1.97: 1 |
| Cu_6_MH | 0.136 | 0.04 | 6.0: 1 | 3.41: 1 |
| Cu_12_MH | 0.271 | 0.04 | 12.0: 1 | 4.60: 1 |
| Cu_24_MH | 0.543 | 0.04 | 24.0: 1 | 4.63: 1 |

Table S2. Detailed primer sequences for qRT-PCR.

| Gene | Forward sequence (5’ to 3’) | Reverse sequence (5’ to 3’) |
| --- | --- | --- |
| IL6 | ACAAGTCCGGAGAGGAGACT | ACAGTGCATCATCGCTGTTC |
| MMP13 | ACCATCCTGTGACTCTTGCG | TTCACCCACATCAGGCACTC |
| MMP3 | GGCTGTGTGCTCATCCTACC | TGGAAAGGTACTGAAGCCAC |
| Col2α1 | TGCTGAAAACCTGGTGATG | GTAACCTCTGTGACCCTTGAC |
| GAPDH | TCCAGTATGACTCTACCCACG | CACGACATACTCAGCACCAG |

Table S3. C, O and Cu contents of CuMHs by XPS and the corresponding surface adsorption properties by BET.

| Symbol | C (%) | O (%) | Cu (%) | Pore size (nm) | Pore volume (cm^3^) | Specific surface area (m^2^/ g) |
| --- | --- | --- | --- | --- | --- | --- |
| Cu_1.5_MH | 67.52 | 29.13 | 3.36 | / | 0.004682 | 0.2743 |
| Cu_3_MH | 59.05 | 32.21 | 8.74 | 9.1397 | 0.515510 | 212.4361 |
| Cu_6_MH | 54.10 | 34.94 | 10.96 | 13.5247 | 0.461252 | 144.1073 |
| Cu_12_MH | 51.03 | 36.01 | 12.95 | 13.1523 | 0.556411 | 102.7756 |
| Cu_24_MH | 43.13 | 38.60 | 18.27 | 28.6901 | 0.586588 | 95.8288 |
